# Preconception paternal ethanol exposures induce alcohol-related craniofacial growth deficiencies in fetal offspring

**DOI:** 10.1172/JCI167624

**Published:** 2023-06-01

**Authors:** Kara N. Thomas, Nimisha Srikanth, Sanat S. Bhadsavle, Kelly R. Thomas, Katherine N. Zimmel, Alison Basel, Alexis N. Roach, Nicole A. Mehta, Yudhishtar S. Bedi, Michael C. Golding

**Affiliations:** Department of Veterinary Physiology and Pharmacology, School of Veterinary Medicine and Biomedical Sciences, Texas A&M University, College Station, Texas, USA.

**Keywords:** Development, Neuroscience, Embryonic development, Epigenetics, Toxicology

**To the Editor:** Fetal alcohol syndrome (FAS) is characterized by a range of structural birth defects, including facial dysmorphia, central nervous system growth deficits (microcephaly), and prenatal/postnatal growth restriction, which correlate with the magnitude of prenatal alcohol exposure (1, 2). Although exclusively attributed to the maternal consumption of alcohol during pregnancy, multiple clinical studies and case reports have emerged describing instances in which infants presenting with alcohol-related birth defects were born to mothers who denied consuming alcohol during pregnancy (2, 3). For example, the Collaboration on FASD Prevalence (CoFASP) research consortium recently conferred a diagnosis of FAS to a cohort of 41 children whose mothers refused to endorse alcohol use during pregnancy (3). The prevailing rationalization for these reported inconsistencies is that the mothers did not faithfully report their prenatal alcohol use (4). However, the recent identification of epigenetic mechanisms of paternal inheritance presents an alternative explanation — that the drinking habits of the birth father may contribute to the emergence of alcohol-related phenotypes in their offspring. Regardless, due to the misconception that sperm do not transmit information beyond the genetic code, the influence of paternal drinking on the development of alcohol-related birth defects has not been rigorously examined.

Due to the development of grossly observable alcohol-induced phenotypes consistent with clinical presentations of FAS, mouse models have become a powerful resource for investigating the development of alcohol-induced dysgenesis (5). Therefore, we developed a multiplex mouse model to test the hypothesis that preconception male alcohol exposures induce alcohol-related craniofacial defects and determine if paternal alcohol use increases the penetrance and severity of alcohol-related phenotypes induced by maternal drinking. Using a 2 × 2 factorial design, we contrasted the emergence of alcohol-induced developmental defects in offspring generated using maternal (MatExp), paternal (PatExp), and dual parental (DualExp) models of alcohol exposure (Figure 1A, Supplemental Methods, and Supplemental Figures 1 and 2; supplemental material available online with this article; https://doi.org/10.1172/JCI167624DS1).

We first employed geometric morphometric analyses (6) to contrast the geometric relationships among 16–18 craniofacial landmarks in offspring derived from control, MatExp, PatExp, and DualExp pairings. Canonical variant analysis of geometric facial relationships revealed that MatExp, PatExp, and DualExp treatments each induced distinct signatures of morphometric change, with paternal exposures frequently inducing the most significant shifts in craniofacial patterning (Figure 1B and Supplemental Figure 3).

Furthermore, our analyses revealed that alcohol-induced changes predominantly centered on the lower portions of the face, including the mandible (lower jaw), maxilla (upper jaw), and positioning of the ear (Figure 1C). As in clinical studies of children with FAS (6), we identified a shift of midline features to the right (Supplemental Figure 3).

To validate our observations, we examined offspring for alterations in established measures of alcohol-induced craniofacial defects (Supplemental Figure 3). Consistent with our geometric morphometric analysis, PatExp and DualExp induced significant reductions in offspring head size (Figure 1D), ocular size (Figure 1E), midfacial depth (Figure 1F), and snout-occipital distance (Figure 1G). Notably, the reductions in ocular size primarily manifested in the right eye (Figure 1E and Supplemental Figure 3).

Finally, we conducted a dose-response analysis, contrasting offspring snout-occipital distance and body weight–normalized brain weights with the parental average daily ethanol dose (g/kg). We identified a significant correlation between both measurements and parental alcohol dose for male offspring across the PatExp and DuelExp treatments (Figure 1, H and I). In contrast, we only observed a significant interaction for female offspring in the MatExp and DualExp treatments (Figure 1, H and I). Aligning with previous studies examining MatExp using the C57BL/6J mouse model (5), our dose-response analyses identified a consistent inflection point of 2.9 g/kg, past which PatExp induced significant craniofacial and central nervous system growth deficiencies (Figure 1, H and I, dashed line).

In 1981, the US Surgeon General issued a public health advisory warning that alcohol use by women during pregnancy could cause birth defects. Since this time, maternal alcohol use during pregnancy has remained the sole explanation for alcohol-related birth defects. In contrast, paternal drinking and lifestyle choices remain unexamined. Using a physiologically relevant mouse model, our studies are the first to our knowledge to demonstrate that male drinking is a plausible yet completely unexamined factor in the development of alcohol-related craniofacial abnormalities and growth deficiencies. Our study demonstrates the critical need to target both parents in prepregnancy alcohol messaging and to expand epidemiological studies to measure the contributions of paternal alcohol use on children’s health.

## Supplementary Material

Supplemental data

## Figures and Tables

**Figure 1:**
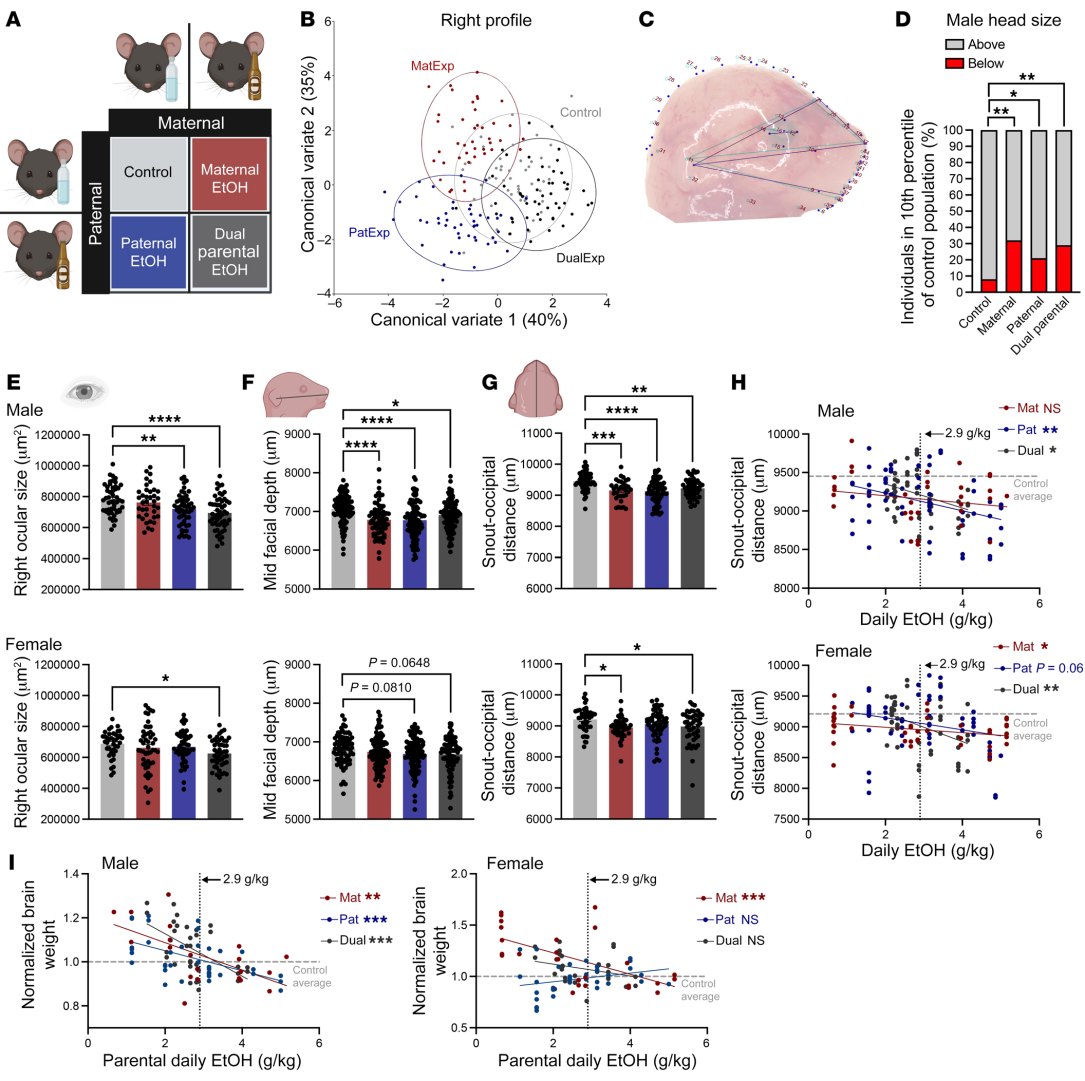
Maternal, paternal, and dual parental alcohol exposures each induce changes in offspring craniofacial patterning. (**A**) We used a 2 × 2 factorial design to contrast craniofacial development among the offspring of maternal (MatExp), paternal (PatExp), and dual parental (DualExp) alcohol exposures. Using geometric morphometrics, followed by canonical variate analysis and multivariate ANOVA, we identified treatment-specific shifts in the profiles of the gestational day 16.5 face (analysis of right profile male offspring shown in **B**, *n* = ~48). (**C**) Representative wire diagram demonstrating the shift in facial landmarks of a PatExp male head along principal component 1. The blue lines demarcate the individual sample, while the turquoise lines demarcate the average of the entire population. (**D**) χ^2^ analysis, followed by Holm-Bonferroni correction, revealed an increased incidence of microcephaly, with an increased proportion of male offspring exhibiting head sizes at or below the smallest 10th percentile of the control population. We used ANOVA, followed by Dunnett’s multiple comparison test, to examine established alcohol-related craniofacial phenotypes, including changes in (**E**) ocular size (right eye shown), (**F**) midfacial depth, and (**G**) snout-occipital distance (males, top; females, bottom; *n* = 30–50). Pearson correlation analysis, followed by Holm-Bonferroni correction, contrasted offspring (**H**) snout-occipital distance (males, top; females, bottom) and (**I**) normalized brain weights (males, left; females, right), with average parental daily EtOH dose (*n* = 30–50, see Supplemental Table 1). Data represent mean ± SEM. *P < 0.05, **P < 0.01, ***P < 0.001, ****P < 0.0001.

## Version 1 04/11/2023

In-Press Preview

## Version 2 06/01/2023

Electronic publication
