# Supplementary material for: Preconception paternal ethanol exposures induce alcohol-related craniofacial growth deficiencies in fetal offspring
Source: J Clin Invest. 2023 Jun 1;133(11):e167624. doi: 10.1172/JCI167624 (PMC10231986; doi:10.1172/JCI167624)
Supplement: Supplemental data [file jci-133-167624-s201.pdf]

Preconception paternal ethanol exposures induce alcohol-related craniofacial growth deficiencies in fetal offspring.

Kara N. Thomas, Nimisha Srikanth, Sanat S. Bhadsavle, Kelly R. Thomas, Katherine N. Zimmel, Alison Basel, Alexis N. Roach, Nicole A. Mehta, Yudhishtar S. Bedi, and Michael C. Golding *

Department of Veterinary Physiology & Pharmacology, School of Veterinary Medicine and Biomedical Sciences, Texas A&M University, College Station, Texas, USA, 77843

* Correspondence:
Michael C. Golding
mgolding@cvm.tamu.edu

Study design

Multiple clinical studies and case reports describe instances where infants diagnosed with Fetal Alcohol Syndrome (FAS) were born to mothers who denied consuming alcohol during pregnancy (1–5). The prevailing explanation offered for these discrepancies is that the mothers lied about their prenatal alcohol use (6). However, these previous studies did not record the alcohol use of the birth father and, therefore, did not adequately consider paternal epigenetic contributions to this pediatric disorder (7). Three of the four diagnostic criteria for FAS include alcohol-induced structural and growth defects (8). Therefore, the objective of this study was to investigate the influences of preconception paternal alcohol consumption on the development of alcohol-related birth defects and determine if paternal alcohol exposures could interact with maternal exposures to exacerbate these outcomes.

Previous studies examining alcohol-induced structural birth defects in rodents have primarily employed oral gavage, a potent inducer of the systemic stress response (9). Stress hormones alter developmental programming in sperm and oocytes, with demonstrated consequences to offspring neurodevelopmental outcomes (10). To avoid this confounder, we utilized a modified version of a voluntary consumption paradigm known as *'Drinking in the Dark'* (11). Using this model, male and female mice consume ethanol (**EtOH**) according to their individual preference, obtaining physiologically relevant plasma alcohol levels while encountering minimal handling. To maximize the clinical relevance of our model, we continuously exposed male mice to EtOH, while in contrast, we only exposed females during an initial preconception period and the first ten days of gestation. This paradigm models the behavior of most women, who cease consumption upon pregnancy diagnosis (12). After establishing our exposure model, we employed a 2x2 factorial experimental design to examine alcohol-related growth and structural birth defects in the offspring of unexposed (Control), maternal- (MatExp), paternal- (PatExp), and dual parental-exposed (DualExp) mice (**Supplemental Figure 1A**). We then assessed established measures of alcohol-induced craniofacial dysgenesis and central nervous system development (13–17) to determine the impacts of each treatment on the emergence of growth and structural birth defects.

Mice

We conducted all experiments under IACUC 2020-0211, approved by the Texas A&M University IACUC. All experiments were performed following IACUC guidelines and regulations, and we report our data per ARRIVE guidelines.

We obtained adult (postnatal day 90) C57BL/6J (Strain #:000664 RRID: IMSR_JAX:000664) mice from the Texas Institute of Genomic Medicine (TIGM) and maintained them in the TIGM facility on a reverse 12-hour light/dark cycle (lights off at 8:30 am) with *ad libitum* access to a standard chow diet (catalog# 2019, Teklad Diets, Madison, WI, United States) and water. To minimize stress, we implemented additional enrichment measures to the animal's home cage, including shelter tubes for males and igloos for females (catalog# K3322 and catalog# K3570, Bio-Serv, Flemington, NJ, United States).

Maternal Periconceptional Exposures and Breeding.

One week before treatment initiation, we acclimated female mice to individual housing conditions. We then randomly assigned postnatal day 90 females to either the experimental (10% w/v ethanol; catalog# E7023; Millipore-Sigma, St. Louis, MO, USA) or Control (water alone) treatments. Then, beginning four hours after the initiation of the dark cycle, we replaced the water bottle of the animal's home cage with an identical bottle containing the appropriate treatment. We maintained these treatments for four hours, then returned the animal's original water bottle. During all experiments, we simultaneously exchanged the water bottles of Control and EtOH-exposed dams to ensure identical conditions. At the end of each week, during their regular cage change, we recorded the weight of each mouse (g) and the amount of fluid consumed (g) and then calculated weekly fluid consumption as grams of fluid consumed per gram of body weight.

We initiated maternal exposures ten days (approximately two estrus cycles) before breeding dams to treated males (Pregestational Day Ten; PGD10; **Supplemental Figure 1A**). After seven to ten days of exposure, we synchronized female reproductive cycles using the Whitten method (18). Then, after the daily Control or EtOH treatment, we placed a single female into the home cage of a treated male. After six hours, we confirmed matings by the presence of a vaginal plug and returned the female mice to their home cage. We ensured males rested for a minimum of 72 hours before the next attempted mating. We subjected dams to minimal handling but maintained the EtOH and Control treatments until gestational day 10.5, when we calculated the change in dam body weight between gestational day Zero and 10.5, then used a body weight gain of approximately 1.8 g as confirmation of pregnancy (19). Upon pregnancy diagnosis, we ceased the Control and EtOH treatments and left females undisturbed until gestational day 16.5. We repeated this procedure until we obtained the requisite number of pregnancies (**Supplemental Table 1**).

Paternal Preconception Ethanol Exposures.

We exposed male mice to alcohol using a prolonged version of the *Drinking in the Dark* paradigm described previously (19–22). At the end of each week, we recorded sire weight (g) and the amount of fluid consumed (g) and then calculated weekly fluid consumption as grams of fluid consumed per gram of body weight. Using methods described by our group (19), we maintained males on the preconception treatments for six weeks, then bred exposed males to treated dams as described above (**Supplemental Figure 1A**).

Exposure Model Implementation and Quantification

During the preconception and pregnancy phases, we did not observe any differences in daily maternal food intake between treatments (**Supplemental Figure 1B**). Moreover, we did not observe any differences in maternal weight gain between treatments (**Supplemental Figure 1C**). Pair-feeding is an additional control employed to account for altered maternal nutrition when drug exposures reduce food intake during pregnancy (23). However, as maternal alcohol exposure did not measurably impact food consumption or maternal weight gain, we did not implement a pair-fed control. During the exposure window of the preconception phase, we did not observe any differences in dam fluid consumption between treatments (**Supplemental Figure 1D**). However, during pregnancy, Control dams consistently drank more fluid (g/kg) during the exposure window than EtOH-exposed dams (**Supplemental Figure 1D**). We did not observe any differences in sire weight gain or fluid consumption between treatments (**Supplemental Figure 1E-F**).

We determined the daily EtOH dose by multiplying the average weekly fluid consumption (g/g) by 0.10 (10% EtOH), divided this number by 7 (days), and converted the resulting values to grams per kilogram (g/kg), consistent with clinical studies (24). We then compared the daily EtOH doses between the preconception and pregnancy phases. Consistent with publications from other groups (11), during the preconception phase, females obtained a significantly higher daily EtOH dose than males (Paternal 2.7 g/kg Maternal: 3.9 g/kg, $p<0.01$, **Supplemental Figure 1G**). However, during the gestational phase (right side of dashed line), pregnant dams obtained a lower daily EtOH dose (2.8 g/kg) and were not significantly different from males (**Supplemental Figure 1G**). Finally, we did not observe any differences in maternal EtOH average daily dose between the MatExp and DualExp treatment groups for either the preconception or gestation phases (preconception MatExp: 3.279 g/kg and Dual Exp 3.011 g/kg; gestation MatExp: 2.945 g/kg and DualExp 2.773 g/kg; **Supplemental Figure 1H**).

Stress during the preconception period modifies the maternally- and paternally-inherited epigenome (10). Therefore, we subjected mice to minimal handling and only measured plasma alcohol concentrations once during the preconception phase. We collected blood from a subset of the treated mice (at the end of the four-hour exposure cycle) and measured plasma alcohol levels using an M1 Alcohol Analyzer (Analox Technologies, Toronto, ON, Canada). During the preconception phase, treated dams exhibited average plasma alcohol levels of 84 mg/dL, while males averaged 92 mg/dL. Plasma alcohol levels were not significantly different between EtOH-treated sires and dams (**Supplemental Figure 1I**). These plasma alcohol levels are entirely consistent with previous studies by our group and others using this model (19, 21, 22, 25). Notably, this concentration is equivalent to blood alcohol levels at or slightly above the U.S. legal limit for operating a motor vehicle (0.084 and 0.092) and is representative of the drinking patterns reported for one-third of U.S. adults (26, 27).

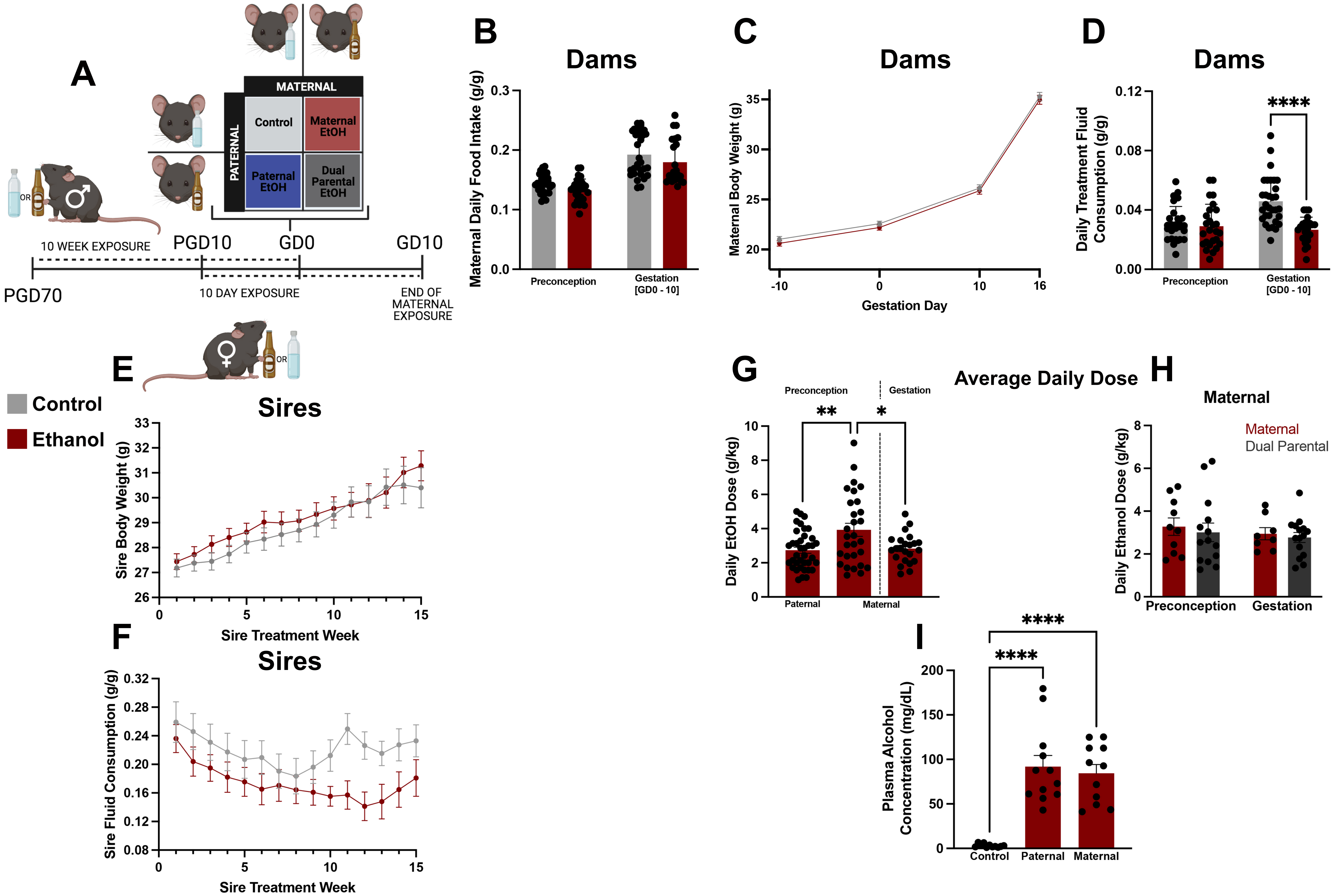
A
MATERNAL
PATERNAL
Control
Maternal EtOH
Paternal EtOH
Dual Parental EtOH
OR
10 WEEK EXPOSURE
PGD10
GD0
GD10
PGD70
10 DAY EXPOSURE
END OF MATERNAL EXPOSURE
B
Dams
Maternal Daily Food Intake (g/g)
Preconception
Gestation [GD0 - 10]
C
Dams
Maternal Body Weight (g)
Gestation Day
D
Dams
Daily Treatment Fluid Consumption (g/g)
****
Preconception
Gestation [GD0 - 10]
E
Control
Ethanol
Sires
Sire Body Weight (g)
Sire Treatment Week
F
Sires
Sire Fluid Consumption (g/g)
Sire Treatment Week
G
Average Daily Dose
Preconception
Gestation
**
*
Daily EtOH Dose (g/kg)
Paternal
Maternal
H
Maternal
Maternal
Dual Parental
Daily Ethanol Dose (g/kg)
Preconception
Gestation
I
****
****
Plasma Alcohol Concentration (mg/dL)
Control
Paternal
Maternal

Supplemental Figure 1. A modified version of the Drinking In The Dark paradigm to study the impacts of maternal, paternal, and dual parental alcohol consumption on offspring health. **A**) Experimental design employed to contrast the impacts of differing patterns of parental alcohol consumption on the emergence of alcohol-related birth defects. **B**) Comparison of daily maternal food intake between the Control and EtOH treatments, both preconception and during gestation. **C**) Comparison of dam weight gain measured across the exposure course and into pregnancy. **D**) Daily maternal fluid consumption between the Control and EtOH treatments during the preconception and gestation phases. Comparisons of sire **E**) weight gain and **F**) fluid consumption across the exposure course. **G**) Average daily dose of EtOH compared between males and females and between the preconception (left of the dashed line) and gestation (right of the dashed line) phases. We calculated the average daily dose by multiplying the average weekly fluid consumption (g/g) by 0.10 (10% EtOH), dividing this number by 7 (days), and converting to g/kg. We used an ANOVA to compare the preconception and pregnancy phases. **H**) Average daily EtOH dose compared between females within the MatExp and DualExp treatments, between the preconception and gestation phases. **I**) Comparison of plasma alcohol levels between treated males and females during the preconception phase. Data represent mean ± SEM, * $P < 0.05$, ** $P < 0.01$, **** $P < 0.0001$.

Tissue Collections and Physiological Measures

Fetal Dissections

On average, males sired offspring after nine to twelve weeks of exposure, and we did not observe any differences between treatment groups (**Supplemental Figure 2A**). Further, we did not observe any significant differences in pregnancy success rates between treatment groups (**Supplemental Figure 2B**). After pregnancy diagnosis, we provided dams with additional cage enrichments, including three nestlets, one Manzanita wood gnawing stick (catalog# W0016, Bio-Serv, Flemington, NJ, USA), and one gummy bone (catalog# K3585, Bio-Serv, Flemington, NJ, USA). On gestational day 16.5, we sacrificed pregnant dams using CO_2 asphyxiation followed by cervical dislocation, then excised the female reproductive tract. We did not observe differences in normalized uterine horn weights or litter size between treatment groups (**Supplementary Figure 2C-D**).

We isolated genomic DNA from the fetal tail using the HotSHOT method (28) and then determined fetal sex using a PCR-based assay described previously (22). We did not observe any differences in the ratio of males and females between treatment groups (**Supplementary Figure 2E**). In clinical studies, FAS children present as small for their gestational age, exhibiting perinatal weights and head circumference below the 10th percentile (29). We did not observe any differences in litter average fetal weights between treatment groups (**Supplementary Figure 2F**). However, in the DualExp treatment group, we did observe an increase in the proportion of male offspring at or below the smallest 10th percentile of the Control population (**Supplementary Figure 2G**) but did not observe this change in female offspring (**Supplementary Figure 2H**). After collecting fetal measures, we imaged offspring heads under a stereomicroscope.

Finally, we selected the four fetuses closest to the cervix from each litter, dissected the fetal brain, and measured brain weights. We observed a significant increase in female brain-to-body weight ratios for offspring in the MatExp and DualExp treatment groups (**Supplementary Figure 2I**). Due to our inability to definitively identify phenotypic sex at this developmental stage and random sampling, there were fewer male fetuses in the MatExp treatment (Male brains: n = 38 control, 18 maternal, 40 paternal, 34 dual parental; **Supplemental Table 1**), which may have limited our ability to detect changes in male offspring brain weights. After dissections, we either fixed tissue samples in 10% neutral buffered formalin (catalog# 16004-128, VWR, Radnor, PA, USA) or snap-froze the tissues on dry ice and stored them at −80°C.

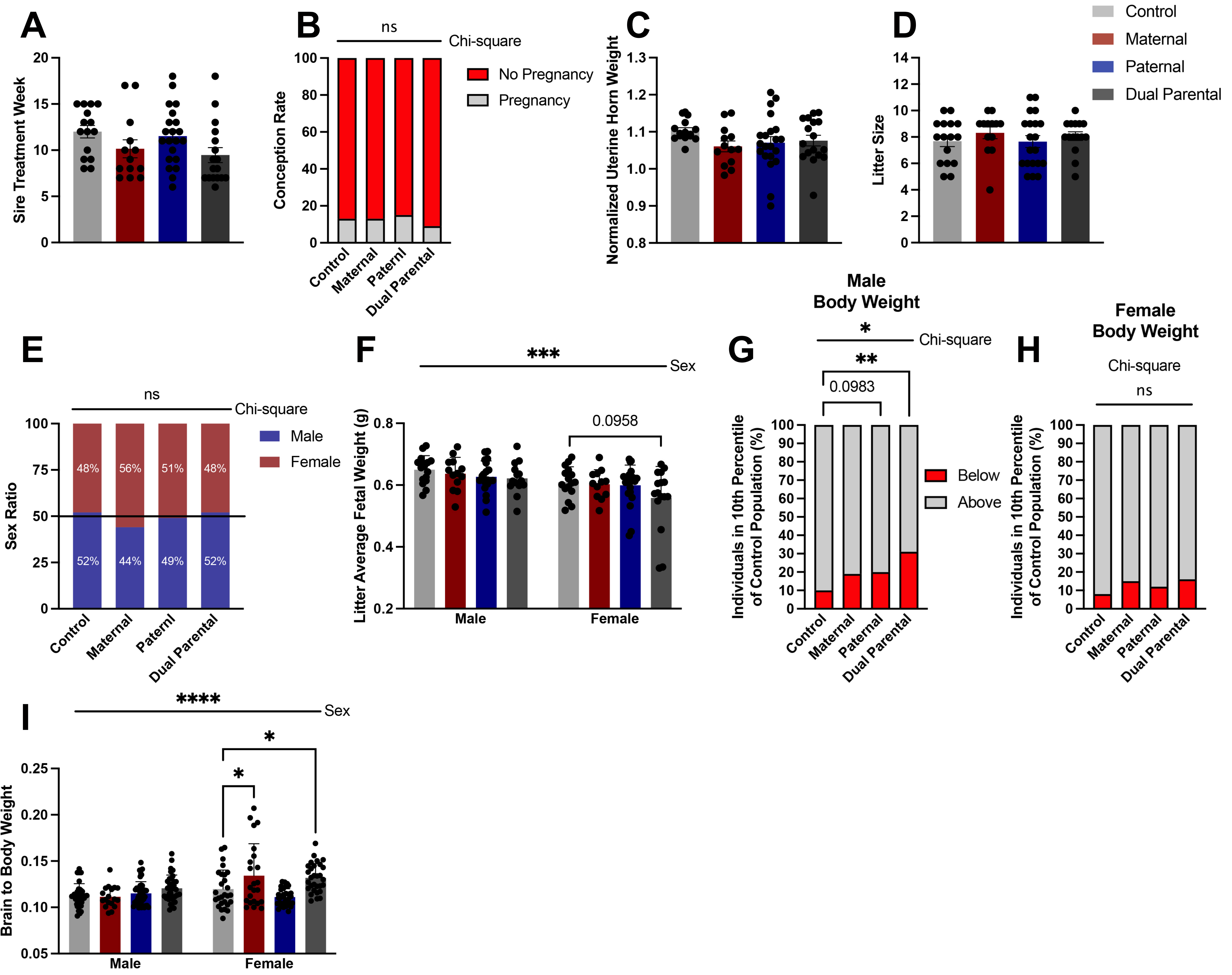
A
Sire Treatment Week
B
ns
Chi-square
Conception Rate
No Pregnancy
Pregnancy
Control
Maternal
Paternl
Dual Parental
C
Normalized Uterine Horn Weight
D
Litter Size
Control
Maternal
Paternal
Dual Parental
E
ns
Chi-square
Sex Ratio
Male
Female
48%
56%
51%
48%
52%
44%
49%
52%
F
***
Sex
Litter Average Fetal Weight (g)
0.0958
Male
Female
Male Body Weight
G
*
Chi-square
**
0.0983
Individuals in 10th Percentile of Control Population (%)
Below
Above
Female Body Weight
H
Chi-square
ns
I
****
Sex
*
*
Brain to Body Weight

Supplemental Figure 2. Analysis of pregnancy and fetal offspring physiological measures. A) Comparison of sire treatment week at the time of conception between treatment groups. **B**) Comparison of pregnancy success rates between treatment groups. We did not detect any differences in **C**) normalized uterine horn weights, **D**) litter size, **E**) the ratio of male and female offspring, or **F**) litter average fetal weights between treatment groups. Differences in the proportion of **G**) male but not **H**) female offspring at or below the smallest 10th percentile of the control population. **I**) Comparison of brain-to-body weight ratios between treatment groups. Data represent mean ± SEM, * $P < 0.05$, ** $P < 0.01$, *** $P < 0.001$, **** $P < 0.0001$.

Craniofacial analysis

2D Imaging

During dissections, we collected 2D images of fetal heads by excising the fetus from the gestational sac and placing the fetus directly under a stereomicroscope (SZX2-ZB10, Olympus, Shinjuku City, Tokyo, Japan) with an attached digital camera (SC-180, Olympus, Shinjuku City, Tokyo, Japan). We used the cellSens Entry software (cellSens Entry Version 3, Olympus, Shinjuku City, Tokyo, Japan) to acquire and analyze 2D images of the frontal and lateral views of fetal heads for further craniofacial analysis.

Geometric morphometrics

FAS is associated with three broad developmental defects: facial dysmorphia, including midline defects and reductions in eye size; central nervous system structural defects, including microcephaly; and prenatal growth restriction (2, 30). Geometric morphometric analysis is a landmark-based analytical technique used to compare the relative positions of facial landmarks and quantify differences in overall biological shape and morphology between populations (31–33). The obtained morphological information includes shape variation, relative shifts in landmark position, differences in feature rotation, and changes in proportional size (31–33). After identifying landmarks, generalized Procrustes analysis (GPA) standardizes all specimens by removing scale from the dataset and minimizing the distance between landmarks using the least squares method (33). This standardization technique then allows the placement of all observed landmark datasets into a common coordinate system. Subsequently, standardized data are examined using canonical variant (CV) analysis to identify the proportional relationships that best distinguish shape differences among groups (31–36). Accordingly, geometric morphometric analysis is widely used, both clinically and experimentally, to study diverse aspects of craniofacial patterning, including the role of enhancers in driving craniofacial development (34), the prevalence of craniofacial phenotypes in genetic syndromes (37) and in characterizing fetal alcohol syndrome-associated craniofacial dysmorphology (15, 16).

We used 12-14 litters per treatment, yielding a sample size of ~48 male and ~48 female offspring per treatment (**Supplemental Table 1**), curated the digital photographs of each fetus within the litter to include their litter ID, sex, and uterine position, then processed images for analysis using the publicly available program **MORPHOJ** (33). First, we used the publicly available program **tpsUtil64** ((38); version 1.82) to generate a TPS database. We then imported collected 2D images of fetal heads into the publicly available image analysis software **tpsDig** ((39) version 3.2) and set the reference scale bar included in the picture to 1 mm. Next, we demarcated 16 landmarks on the left/right profile and 18 landmarks on the front profile, following previously established criteria (32, 40) and morphological landmarks described by Anthony et al., 2010 (13). The employed landmarks included:

Left/Right Photograph Landmark Key

1. Tip of nose
2. Nasion (in between eyes)
3. Top of head (highest point of elevation of head)
4. Curve of skull (cusp of where the skull begins to curve downward)
5. Back of skull (Before projection of spinal column, directly behind the ear)
6. Bottom of mandible (lowest part of the jaw)
7. Front of mandible (Closest to mouth opening, furthest point facing out)
8. Upper philtrum (upper lip, closest point below base of nose)
9. Inner mouth (the innermost corner of the mouth)
10. Edge of snout (Light coloration, where the whiskers would end)
11. Central auditory canal (middle of light-colored tissue)
12. 3 O'clock position of eye (closest to the auditory canal, exterior of eye)
13. 12 O'clock position of eye (towards top of head, exterior of eye)
14. 9 O'clock position of eye (closest to the snout, exterior of eye)
15. 6 O'clock position of eye (bottom most part of eye, closest to the jaw)
16. Pupil (center of eye)

Front Photograph Landmark Key

1. Top of head (Central, highest point of head, in line with the nose)
2. Bottom of mandible (Bottom-most point of jaw)
3. Right corner of mouth (Furthest right point where mouth closes)
4. Left corner of mouth (Furthest left point where mouth closes)
5. Top of philtrum (Closest point below the base of nose)
6. Bottom of philtrum (Ventral extent of philtrum, closest junction between two lips)
7. Tip of nose (top, central most part of the nose)
8. Nasion (Central point between the two eyes)
9. 3 O'clock position of left eye (medial-most part of the external eye)
10. 12 O'clock position of left eye (topmost part of the external eye)
11. 9 O'clock position of left eye (medial-most part of the external eye)
12. 6 O'clock position of left eye (bottom most part of external eye)
13. Pupil of left eye (Center of the left eye, lighter color)
14. 3 O'clock position of right eye (medial-most part of the external eye)
15. 12 O'clock position of right eye (topmost part of the external eye)
16. 9 O'clock position of right eye (medial-most part of the external eye)
17. 6 O'clock position of right eye (bottom most part of external eye)
18. Pupil of right eye (Center of the right eye, lighter color)

To ensure consistency, a single individual (N.S.) demarcated the same landmarks in the exact location and order for each image. We then created a linear outline around the head and digitized the landmarks and outlines as a TPS file. **tpsDig** (39) then adds additional landmarks, including the midpoints between features and other aspects of the outline, for a total of 47 landmarks for the front profile and 45 for the left/right. We then imported the TPS files into the **MORPHOJ** software (33) (software version build 1.07a, Java version 1.8.0_291 (Oracle Corporation)) to conduct geometric morphometric analysis. We added classifiers describing each treatment group, then separately normalized male and female left, right, and frontal datasets for scale, rotation, and translation using the Procrustes fit feature (33). We then generated a covariance matrix, which we used to conduct Principal Component Analysis (PCA). Our PCA analysis revealed that PC1 and PC2 described most (Males Front 68.5%, Left 71.0%, and Right 68.5%; Females Front 69.6%, Left 69.0%, and Right 69.6%) of the variation in our model.

We then used Canonical Variate (CV) analysis to identify differences in facial features between treatments and exported the raw CV scores into the Paleontological Statistics Software Package for Education and Data Analysis (PAST) analysis software ((41) version 4.03; [https://past.en.lo4d.com/windows]). Next, we conducted multivariate analyses of the raw CV scores using statistical methods described previously (32, 34–36). These included the parametric Multivariate analysis of variance (MANOVA) and non-parametric Analysis of similarities (ANOSIM), and Permutational multivariate analysis of variance (PERMANOVA) tests, followed by Bonferroni correction to identify significant differences in clustering and distance between treatment groups. Finally, we generated the CV lollipop diagrams and scatter plots using the graphing features of MORPHOJ (33).

Our multivariate analysis of male CV scores revealed significant differences between treatments for the front (MANOVA p = 2.223E-90, ANOSIM p<0.0001, R = 0.6316, PERMANOVA p<0.0001; **Supplemental Figure 3A),** left (MANOVA p = 4.832E-67, ANOSIM p<0.0001, R = 0.4736, PERMANOVA p<0.0001; **Supplemental Figure 3B),** and right profiles (MANOVA p = 6.737E-87, ANOSIM p<0.0001, R = 0.6178, PERMANOVA p<0.0001; **Supplemental Figure 3C),** with each treatment exhibiting significant differences during pairwise comparisons (**Supplemental Table 1**). Notably, Procrustes ANOVA identified significant (p = 0.0103) shifts in overall face shape between treatments. Further analyses of male craniofacial shape revealed that most changes centered around the lower portion of the face, including the mandible (lower jaw), maxilla (upper jaw), and positioning of the ear (**Supplemental Figure 3D-E**). As in clinical studies of FAS children (16), we identified a shift of midline landmarks to the right (**Supplemental Figure 3D**).

Our multivariate analysis of female CV scores revealed significant differences between treatments in the front (MANOVA p = 7.85E-95, ANOSIM p<0.0001, R = 0.6848, PERMANOVA p<0.0001; **Supplemental Figure 3F)** left (MANOVA p = 2.89E-77, ANOSIM p<0.0001, R = 0.5696, PERMANOVA p<0.0001; **Supplemental Figure 3G**), and right profiles (MANOVA p = 1.15E-71, ANOSIM p<0.0001, R = 0.5196, PERMANOVA p<0.0001; **Supplemental Figure 3H)**, with each treatment exhibiting significant differences during pairwise comparisons (**Supplemental Table 1**). As with male offspring,

most identified changes centered around the lower portion of the face (**Supplemental Figure 3I-J**). However, Procrustes ANOVA did not identify any significant shifts in female overall face shape between treatments.

Linear morphometrics

To validate our morphometric analyses, we conducted linear measurements of fetal craniofacial features using 2D images and the analysis software cellSens Entry (cellSens Entry Version 3, Olympus, Shinjuku City, Tokyo, Japan). We used the length tool on lateral images to measure upper facial depth, midfacial depth, and lower facial depth, following previously described facial landmarks (13). Similar to clinical studies (42), we determined ear size and positioning by measuring the distance from the dorsal to ventral aspects of the external ear and the distance between the central auditory canal and the center of the pupil, respectively (**Supplemental Figure 3K**). For male offspring, we identified reductions in upper facial depth in the PatExp treatment and a trend towards reduced size in the MatExp treatment ($p = 0.0617$) (**Supplemental Figure 3L**). We did not observe any differences in the upper facial depth of female offspring across any treatment (**Supplemental Figure 3L)**. Consistent with our geometric morphometric analysis, male offspring across all treatments displayed significant reductions in midfacial and lower facial depth, while female offspring in the PatExp and DualExp treatments displayed similar trends ($p = 0.08$ to 0.06) (**Figure 1F; Supplemental Figure 3M**). Male and female offspring across all treatment groups exhibited significant reductions in ear size (**Supplemental Figure 3N**). Further, male offspring across all treatments and DualExp female offspring exhibited significant decreases in the distance between the central aspects of the eyes and ears (**Supplemental Figure 3O**).

In clinical studies, alcohol-induced changes in craniofacial shape exhibit dose-dependent effects (2). Therefore, we used linear regression analysis to compare changes in offspring shape with the parental average daily EtOH dose (g/kg). These analyses revealed dose-dependent effects on male snout-occipital distance, midfacial depth, and body weight normalized brain weights across most treatments (**Figure 1 H-I; Supplemental Figure 3P**). Female offspring only displayed dose-dependent effects in a subset of these measures.

We then used the area tool on lateral images to determine ocular size (area um^2), then used an ANOVA to compare the impacts of each treatment. Male offspring in the PatExp and DualExp treatments exhibited significant reductions in ocular size, while only DualExp females were significantly different from the controls (**Supplemental Figure 3Q**). Previous studies examining EtOH-induced craniofacial dysgenesis using mouse models of maternal exposure demonstrate that reductions in eye size appear right-side dominant (17, 43). Similarly, clinical studies demonstrate that gestational EtOH exposures alter facial symmetry with a right-shift in facial features (16). Consistent with these previous studies, we observed significant reductions in ocular size for PatExp and DualExp male and DualExp female offspring in the right but not the left eye (**Figure 1E**, **Supplemental Figure 3R-S**).

We used the cellSens Entry length tool on frontal images to determine philtrum length, snout-occipital distance, inner canthal distance, and biparietal distance, measuring the latter across the axis of the eyes, as previously described (13). We did not observe any significant differences in philtrum length, although female offspring in the PatExp treatment trended towards a reduction ($p = 0.0706$) (data not shown). Male offspring across all treatment groups exhibited reductions in snout-occipital distance, while only female offspring in the MatExp and DualExp treatment groups exhibited significant reductions (**Figure 1G**). Male offspring in the PatExp and DualExp treatments exhibited reduced inner canthal distances, while female offspring did not exhibit any significant changes in this feature (**Supplemental Figure 3T**). Male offspring across all treatment groups exhibited reductions in biparietal distance, while only female offspring in the DualExp treatment exhibited reductions (**Supplemental Figure 3U**). Finally, to determine if changes in inner canthal distance are driven by microcephaly or represent relative changes in eye spacing, we normalized inner canthal distance to biparietal distance and compared offspring between treatment groups. Male and female offspring from all three treatment groups exhibited increases in relative inner canthal distance (**Supplemental Figure 3V**).

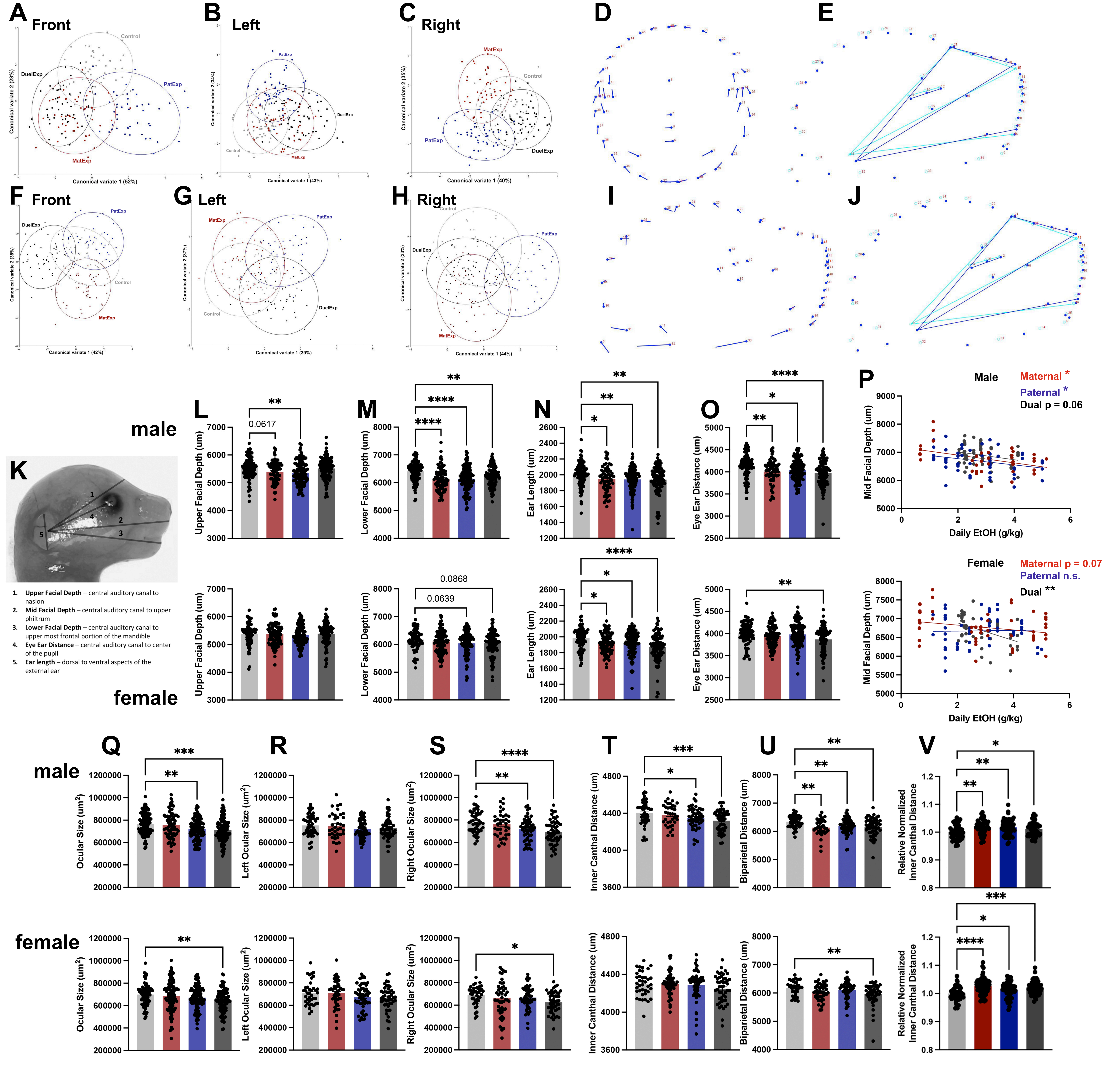
A Front
B Left
C Right
D
E
F Front
G Left
H Right
I
J
Control
DuelExp
PatExp
MatExp
Canonical variate 1 (52%)
Canonical variate 2 (28%)
Canonical variate 1 (43%)
Canonical variate 2 (34%)
Canonical variate 1 (40%)
Canonical variate 2 (35%)
Canonical variate 1 (42%)
Canonical variate 2 (38%)
Canonical variate 1 (39%)
Canonical variate 2 (37%)
Canonical variate 1 (44%)
Canonical variate 2 (33%)
K
1. Upper Facial Depth – central auditory canal to nasion
2. Mid Facial Depth – central auditory canal to upper philtrum
3. Lower Facial Depth – central auditory canal to upper most frontal portion of the mandible
4. Eye Ear Distance – central auditory canal to center of the pupil
5. Ear length – dorsal to ventral aspects of the external ear
male
female
L
M
N
O
P
Upper Facial Depth (um)
Lower Facial Depth (um)
Ear Length (um)
Eye Ear Distance (um)
Mid Facial Depth (um)
Daily EtOH (g/kg)
0.0617
0.0868
0.0639
Male
Maternal *
Paternal *
Dual p = 0.06
Female
Maternal p = 0.07
Paternal n.s.
Dual **
Q
R
S
T
U
V
Ocular Size (um²)
Left Ocular Size (um²)
Right Ocular Size (um²)
Inner Canthal Distance (um)
Biparietal Distance (um)
Relative Normalized Inner Canthal Distance

Supplemental Figure 3 Maternal, Paternal, and Dual Parental alcohol exposures program dose-dependent changes in offspring craniofacial patterning. We conducted geometric morphometric analysis on the front, left, and right profile images obtained from ~48 male and ~48 female offspring per treatment, then employed canonical variate analysis to identify significant differences in clustering and distance between treatment groups. We identified significant shifts in the front (**A, F**), left (**B, G**), and right (**C, H**) profiles of male and female offspring. Morphometrics revealed a right-shift in central facial features and a rear shift in the lower jaw. Lollipop diagram describing the shift in central (male offspring **D**) and lower (female offspring **I**) facial features along PC1, with the stick of the lollipop signifying the shift further along the PC1 axis. Wire diagrams demonstrating the shift in key facial landmarks along PC1 (male offspring **E,** female offspring **J**), with the turquoise line demarcating the average and the blue line demarcating the shift. To validate our morphometric analysis, we assayed established measures of craniofacial morphology (**K**) disrupted in mouse models of prenatal alcohol exposure (13) and compared the effects of each treatment on male (row top) and female (row bottom) offspring. We measured the effects of parental alcohol exposures on upper facial depth (**L**), lower facial depth (**M**), ear length (**N**), and the distance between the central auditory canal and the center of the pupil (**O**). We then conducted a Pearson correlation analysis contrasting the midfacial depth of male (top) and female (bottom) offspring with average parental daily EtOH dose (**P**). We measured the effects of parental alcohol exposures on both eyes (**Q**), then individually examined the left (**R**) and right (**S**) eyes in male (row top) and female (row bottom) offspring. We measured the effects of parental alcohol exposures on inner canthal (**T**) and biparietal (**U**) distance in male (row top) and female (row bottom) offspring. We normalized inner canthal distance to biparietal (**V**) and compared the effects of each treatment on male and female offspring. Data represent mean ± SEM, * $P < 0.05$, ** $P < 0.01$, *** $P = 0.001$, **** $P = 0.001$.

Data handling and statistical analysis

We subjected all data generated during this study to a detailed data management plan that prioritizes safe and efficient data handling that allows long-term storage, retrieval, and preservation. We recorded Initial measures by hand, then inserted these into Google Sheets or Microsoft Excel for downstream analysis using GraphPad Prism 8 (RRID:SCR_002798, GraphPad Software Inc., La Jolla, CA, USA). We analyzed all data sets with statistical significance set at alpha = 0.05, then employed the ROUT test (Q = 1%) to identify outliers. Next, we verified the normality of the datasets using the Shapiro–Wilk test and verified equal variance using the Brown-Forsythe test. If data passed normality and variance testing (alpha = 0.05), we employed either a One-way or Two-way ANOVA or an unpaired, parametric (two-tailed) t-test. If the data failed the test for normality or we observed unequal variance, we ran a Kruskal-Wallis test followed by Dunn's multiple comparisons test or a non-parametric Mann–Whitney test.

For measures of fetal weight, we determined the male and female average for each litter and used this value as the individual statistical unit. Subsequently, we identified the tenth percentile fetal weight for the Control population, determined the proportion of offspring above and below this value, then ran a Chi-square analysis to compare the proportions between treatments. For the analysis of fetal brain weights, we selected the four fetuses closest to the cervix from each litter. We present detailed descriptions of each statistical test, sample size, and resulting p-values in **Supplemental Table 1**.

Table 1: Descriptions of the statistical tests for each figure.

GRAPH	STATISTICAL TEST	SAMPLE SIZE
**Figure 1. Maternal, Paternal, and Dual Parental alcohol exposures each induce changes in offspring craniofacial patterning**.		
**b.** Male right profile canonical variant analysis	Canonical variant analysis	n = 50 control, 47 maternal, 47 paternal, 48 dual parental
**c.** Male right profile wire diagram shift in facial features	MANOVA (Wilk's Lambda Statistic), ANOSIM, PERMANOVA	n = 50 control, 47 maternal, 47 paternal, 48 dual parental
**d.** Males with head size below the 10th percentile of the control population	Chi-square test followed by Holm-Bonferroni correction	n = 61 control, 37 maternal, 66 paternal, 62 dual parental
**e.** Right ocular size (top: male, bottom: female)	One-way ANOVA, Dunnett's multiple comparison test	Male: n = 52 control, 37 maternal, 57 paternal, 53 dual parental; Female: n = 39 control, 49 maternal, 54 paternal, 48 dual parental
**f.** Mid facial depth (top: male, bottom: female)	One-way ANOVA, Dunnett's multiple comparison test	Male: n = 103 control, 72 maternal, 111 paternal, 106 dual parental; Female: n = 76 control, 98 maternal, 107 paternal, 96 dual parental
**g.** Snout-occipital distance (top: male, bottom: female)	One-way ANOVA, Dunnett's multiple comparison test	Male: n = 53 control, 33 maternal, 63 paternal, 52 dual parental; Female: n = 40 control, 45 maternal, 57 paternal, 48 dual parental
**h.** Snout-occipital distance dose correlation	Pearson correlation, two-tailed, followed by Holm-Bonferroni correction	Males: n = 33 maternal, 63 paternal, 52 dual parental; Females: n = 28 maternal, 35 paternal, 30 dual parental

**Sex**	**Treatment**	**r**	**R^2**	**p-value**	**Summary**
Males	Maternal	-0.1926	0.0371	0.2829	ns
	Paternal	-0.3695	0.1365	0.0029	**
	Dual	-0.3447	0.1188	0.0123	*
Females	Maternal	-0.3447	0.1188	0.0219	*
	Paternal	-0.2447	0.0599	0.0655	#
	Dual	-0.3855	0.1486	0.0074	**

**i.** Normalized brain weights dose correlation	Pearson correlation, two-tailed, followed by Holm-Bonferroni correction	Males: n = 20 maternal, 38 paternal, 32 dual parental; Females: n = 28 maternal, 35 paternal, 30 dual parental

Sex	Treatment	r	R^2	p-value	Summary
Males	Maternal	-0.6114	0.3738	0.0042	**
	Paternal	-0.5466	0.2988	0.0004	***
	Dual	-0.5612	0.3150	0.0008	***
Females	Maternal	-0.6365	0.4051	0.0003	***
	Paternal	0.2870	0.0824	0.0946	#
	Dual	-0.2940	0.0865	0.1148	ns

Supplemental Figure 1. A modified version of the Drinking In The Dark paradigm to study the impacts of maternal, paternal, and dual parental alcohol consumption on offspring health.

**b.** Maternal daily food intake<br>**c.** Maternal body weight<br>**d.** Maternal daily treatment fluid consumption	Two-way ANOVA, Šídák's multiple comparisons test	Preconception: n = 27 control, 27 ethanol; Gestation: n = 30 control, 23 ethanol
**e.** Paternal body weight<br>**f.** Paternal fluid consumption	Two-way ANOVA, Šídák's multiple comparisons test	n = 23 control, 24 ethanol
**g.** Paternal and maternal daily ethanol dose	Two-way ANOVA, Tukey’s multiple comparisons test	n = 24 paternal ethanol, 27 preconception maternal ethanol, 23 gestation maternal ethanol
**h.** Maternal daily ethanol dose	Two-way ANOVA, Šídák's multiple comparisons test	Preconception: 10 maternal, 14 dual parental; Gestation: 8 maternal, 15 dual parental
**i.** Paternal and maternal plasma alcohol concentration	Two-way ANOVA, Tukey’s multiple comparisons test	n = 11 control, 12 paternal ethanol, 11 maternal ethanol

Supplemental Figure 2. Analysis of pregnancy and fetal offspring physiological measures.

**a.** Paternal treatment week at time of conception	One-way ANOVA, Tukey’s multiple comparison test	n = 15 control, 13 maternal, 21 paternal, 17 dual parental

**b.** Conception rate	Chi-square test	n = 136 control, 125 maternal, 135 paternal, 191 dual parental
**c.** Normalized uterine horn weight<br>**d.** Litter size	One-way ANOVA, Tukey’s multiple comparison test	n = 16 control, 13 maternal, 20 paternal, 16 dual parental
**e.** Sex ratio	Chi-square test	n = control: 64 males, 59 females; maternal: 47 males, 61 females; paternal: 71 males, 75 females; dual parental: 65 males, 61 females
**f.** Litter average fetal weight	Two-way ANOVA, Tukey’s multiple comparison test	Male: n = 16 control, 13 maternal, 19 paternal, 14 dual parental; Female: n = 16 control, 12 maternal, 20 paternal, 16 dual parental
**g.** Males with body weight below the 10th percentile of the control population<br>**h.** Females with body weight below the 10th percentile of the control population	Chi-square test, followed by Holm-Bonferroni correction	Male: n = 63 control, 47 maternal, 71 paternal, 65 dual parental; Female: n = 59 control, 61 maternal, 75 paternal, 61 dual parental
**i.** Male and female brain to body weight ratio	Two-way ANOVA, Tukey’s multiple comparison test	Male: n = 38 control, 18 maternal, 40 paternal, 34 dual parental; Female: n = 27 control, 23 maternal, 32 paternal, 30 dual parental
**Supplemental Figure 3. Maternal, Paternal, and Dual Parental alcohol exposures program dose-dependent changes in offspring craniofacial patterning.**		
**a-c.** Male front, left, and right profile canonical variant analysis<br>**f-h.** Female front, left, and right profile canonical variant analysis	Canonical variant analysis	Males: n = 50 control, 47 maternal, 47 paternal, 48 dual parental; Female: n = 46 control, 49 maternal, 41 paternal, 54 dual parental

a. Male front profile: MANOVA p = 2.223E-90, ANOSIM p<0.0001, R = 0.6316, PERMANOVA p<0.0001

**MANOVA**	Control	Maternal	Paternal	Dual
Control		5.29E-22	1.57E-24	2.87E-23
Maternal	5.29E-22		5.12E-26	2.53E-18
Paternal	1.57E-24	5.12E-26		4.26E-30
Dual	2.87E-23	2.53E-18	4.26E-30	

**ANOSIM**	Control	Maternal	Paternal	Dual
Control		0.0006	0.0006	0.0006
Maternal	0.0006		0.0006	0.0006
Paternal	0.0006	0.0006		0.0006
Dual	0.0006	0.0006	0.0006	

**PERMANOVA**	Control	Maternal	Paternal	Dual
Control		0.0006	0.0006	0.0006
Maternal	0.0006		0.0006	0.0006
Paternal	0.0006	0.0006		0.0006
Dual	0.0006	0.0006	0.0006	

b. Male left profile: MANOVA p = 4.832E-67, ANOSIM p<0.0001, R = 0.4736, PERMANOVA p<0.0001

MANOVA	Control	Maternal	Paternal	Dual	ANOSIM	Control	Maternal	Paternal	Dual	PERMANOVA	Control	Maternal	Paternal	Dual
Control		5.05E-17	2.35E-20	6.92E-24	Control		0.0006	0.0006	0.0006	Control		0.0006	0.0006	0.0006
Maternal	5.05E-17		1.50E-17	6.96E-17	Maternal	0.0006		0.0006	0.0006	Maternal	0.0006		0.0006	0.0006
Paternal	2.35E-20	1.50E-17		6.42E-21	Paternal	0.0006	0.0006		0.0006	Paternal	0.0006	0.0006		0.0006
Dual	6.92E-24	6.96E-17	6.42E-21		Dual	0.0006	0.0006	0.0006		Dual	0.0006	0.0006	0.0006	

c. Male right profile: MANOVA p = 6.737E-87, ANOSIM p<0.0001, R = 0.6178, PERMANOVA p<0.0001

MANOVA	Control	Maternal	Paternal	Dual	ANOSIM	Control	Maternal	Paternal	Dual	PERMANOVA	Control	Maternal	Paternal	Dual
Control		2.82E-2	2.07E-21	1.12E-20	Control		0.0006	0.0006	0.0006	Control		0.0006	0.0006	0.0006
Maternal	2.07E-21		6.43E-23	2.92E-24	Maternal	0.0006		0.0006	0.0006	Maternal	0.0006		0.0006	0.0006
Paternal	2.82E-23	6.43E-23		3.12E-25	Paternal	0.0006	0.0006		0.0006	Paternal	0.0006	0.0006		0.0006
Dual	1.12E-20	2.92E-24	3.12E-25		Dual	0.0006	0.0006	0.0006		Dual	0.0006	0.0006	0.0006	

f. Female front profile: MANOVA p = 7.85E-95, ANOSIM p<0.0001, R = 0.6848, PERMANOVA p<0.0001

MANOVA	Control	Maternal	Paternal	Dual	ANOSIM	Control	Maternal	Paternal	Dual	PERMANOVA	Control	Maternal	Paternal	Dual
Control		1.55E-2	1.01E-18	3.75E-25	Control		0.0006	0.0006	0.0006	Control		0.0006	0.0006	0.0006
Maternal	9.29E-26		9.29E-26	8.57E-29	Maternal	0.0006		0.0006	0.0006	Maternal	0.0006		0.0006	0.0006
Paternal	1.01E-18	9.29E-26		2.04E-2	Paternal	0.0006	0.0006		0.0006	Paternal	0.0006	0.0006		0.0006
Dual	3.75E-2	8.57E-29	2.04E-27		Dual	0.0006	0.0006	0.0006		Dual	0.0006	0.0006	0.0006	

g. Female left profile: MANOVA p = 2.89E-77, ANOSIM p<0.0001, R = 0.5696, PERMANOVA p<0.0001

MANOVA	Control	Maternal	Paternal	Dual	ANOSIM	Control	Maternal	Paternal	Dual	PERMANOVA	Control	Maternal	Paternal	Dual
Control		2.64E-21	3.49E-22	2.21E-24	Control		0.0006	0.0006	0.0006	Control		0.0006	0.0006	0.0006

Maternal	5.29E-22		9.38E-21	1.42E-18	Paternal	0.0006		0.0006	0.0006	Paternal	0.0006		0.0006	0.0006
Paternal	1.57E-24	9.38E-21		1.23E-18	Maternal	0.0006	0.0006		0.0006	Maternal	0.0006	0.0006		0.0006
Dual	2.87E-23	1.42E-18	1.23E-18		Dual	0.0006	0.0006	0.0006		Dual	0.0006	0.0006	0.0006	
**h.** Female right profile: MANOVA p = 1.15E-71, ANOSIM p<0.0001, R = 0.5196, PERMANOVA p<0.0001														
**MANOVA**	Control	Maternal	Paternal	Dual	**ANOSIM**	Control	Maternal	Paternal	Dual	**PERMANOVA**	Control	Maternal	Paternal	Dual
Control		1.73E-20	5.44E-19	5.69E-20	Control		0.0006	0.0006	0.0006	Control		0.0006	0.0006	0.0006
Maternal	1.73E-20		5.83E-17	5.19E-20	Maternal	0.0006		0.0006	0.0006	Maternal	0.0006		0.0006	0.0006
Paternal	5.44E-19	5.83E-17		2.54E-21	Paternal	0.0006	0.0006		0.0006	Paternal	0.0006	0.0006		0.0006
Dual	5.69E-20	5.19E-20	2.54E-21		Dual	0.0006	0.0006	0.0006		Dual	0.0006	0.0006	0.0006	

**d.** Male frontal profile lollipop diagram	MANOVA (Wilk's Lambda Statistic), ANOSIM, PERMANOVA	n = 50 control, 47 maternal, 47 paternal, 48 dual parental
**i.** Female right profile lollipop diagram shift in facial features	MANOVA (Wilk's Lambda Statistic), ANOSIM, PERMANOVA	Female: n = 46 control, 49 maternal, 41 paternal, 54 dual parental
**e.** Male right profile wire diagram<br>**j.** Female right profile wire diagram	MANOVA (Wilk's Lambda Statistic), ANOSIM, PERMANOVA	Males: n = 50 control, 47 maternal, 47 paternal, 48 dual parental; Female: n = 46 control, 49 maternal, 41 paternal, 54 dual parental
**l.** Upper facial depth (top: male, bottom: female)	One-way ANOVA, Dunnett's multiple comparison test	Males: n = 103 control, 73 maternal, 111 paternal, 106 dual parental; Female: n = 78 control, 98 maternal, 108 paternal, 96 dual parental
**m.** Lower facial depth (top: male, bottom: female)	One-way ANOVA, Dunnett's multiple comparison test	Males: n = 103 control, 74 maternal, 111 paternal, 106 dual parental; Female: n = 78 control, 98 maternal, 108 paternal, 96 dual parental
**n.** Ear length (top: male, bottom: female)	One-way ANOVA, Dunnett's multiple comparison test	Males: n = 104 control, 65 maternal, 124 paternal, 105 dual parental; Female: n = 78 control, 90 maternal, 116 paternal, 94 dual parental
**o.** Eye ear distance (top: male, bottom: female)	One-way ANOVA, Dunnett's multiple comparison test	Males: n = 104 control, 66 maternal, 124 paternal, 106 dual parental; Female: n = 78 control, 90 maternal, 116 paternal, 95 dual parental

**p.** Mid facial depth dose correlation (top: male, bottom: female)	Pearson correlation, two-tailed, followed by Holm-Bonferroni correction		Males: n = 37 maternal, 55 paternal, 53 dual parental; Females: n = 49 maternal, 53 paternal, 47 dual parental		
**Sex**	**Treatment**	**r**	**R^2**	**p-value**	**Summary**
Males	Maternal	-0.4160	0.1731	0.0104	*
	Paternal	-0.3076	0.0946	0.0223	*
	Dual	-0.2561	0.0656	0.0641	#
Females	Maternal	-0.2535	0.0642	0.0789	#
	Paternal	-0.0249	0.0006	0.8594	ns
	Dual	-0.4414	0.1948	0.0019	**
**q.** Ocular size (top: male, bottom: female)	One-way ANOVA, Dunnett's multiple comparison test		Males: n = 104 control, 74 maternal, 114 paternal, 106 dual parental; Female: n = 78 control, 99 maternal, 108 paternal, 96 dual parental		
**r.** Left ocular size (top: male, bottom: female)	One-way ANOVA, Dunnett's multiple comparison test		Male: n = 52 control, 37 maternal, 57 paternal, 53 dual parental; Female: n = 39 control, 50 maternal, 54 paternal, 48 dual parental		
**s.** Right ocular size (top: male, bottom: female)	One-way ANOVA, Dunnett's multiple comparison test		Male: n = 52 control, 37 maternal, 57 paternal, 53 dual parental; Female: n = 39 control, 49 maternal, 54 paternal, 48 dual parental		
**t.** Inner canthal distance (top: male, bottom: female)	One-way ANOVA, Dunnett's multiple comparison test		Male: n = 52 control, 37 maternal, 53 paternal, 53 dual parental; Female: n = 39 control, 50 maternal, 53 paternal, 48 dual parental		
**u.** Biparietal distance (top: male, bottom: female)	One-way ANOVA, Dunnett's multiple comparison test		Male: n = 53 control, 33 maternal, 63 paternal, 52 dual parental; Female: n = 40 control, 49 maternal, 57 paternal, 48 dual parental		
**v.** Relative normalized inner canthal distance (top: male, bottom: female)	One-way ANOVA, Dunnett's multiple comparison test		Male: n = 52 control, 33 maternal, 53 paternal, 52 dual parental; Female: n = 39 control, 49 maternal, 53 paternal, 48 dual parental		

Bibliography

1. Bandoli G et al. Trajectories of prenatal alcohol exposure and behavioral outcomes: Findings from a community-based sample. *Drug Alcohol Depend* 2022;233:109351.

2. Suttie M et al. Facial Dysmorphism Across the Fetal Alcohol Spectrum. *Pediatrics* 2013;131(3):e779–e788.

3. May PA et al. Epidemiology of FASD in a Province in Italy: Prevalence and Characteristics of Children in a Random Sample of Schools. *Alcoholism: Clinical and Experimental Research* 2006;30(9):1562–1575.

4. May PA et al. Maternal risk factors for fetal alcohol syndrome and partial fetal alcohol syndrome in South Africa: a third study.. *Alcohol Clin Exp Res* 2008;32(5):738–753.

5. Eichler A et al. Did you drink alcohol during pregnancy? Inaccuracy and discontinuity of women's self-reports: On the way to establish meconium ethyl glucuronide (EtG) as a biomarker for alcohol consumption during pregnancy. *Alcohol* 2016;54:39–44.

6. Jacobson SW, Chiodo LM, Sokol RJ, Jacobson JL. Validity of maternal report of prenatal alcohol, cocaine, and smoking in relation to neurobehavioral outcome. *Pediatrics* 2002;109(5):815–826.

7. Olshan AF, Faustman EM. Male-Mediated Developmental Toxicity. *Annual Review of Public Health* 1993;14(1):159–181.

8. Hoyme HE et al. Updated Clinical Guidelines for Diagnosing Fetal Alcohol Spectrum Disorders. *Pediatrics* 2016;138(2):e20154256.

9. Brown AP, Dinger N, Levine BS. Stress produced by gavage administration in the rat.. *Contemp Top Lab Anim Sci* 2000;39(1):17–21.

10. Chan JC, Nugent BM, Bale TL. Parental Advisory: Maternal and Paternal Stress Can Impact Offspring Neurodevelopment. *Biol Psychiatry* 2018;83(10):886–894.

11. Rhodes JS, Best K, Belknap JK, Finn DA, Crabbe JC. Evaluation of a simple model of ethanol drinking to intoxication in C57BL/6J mice.. *Physiol Behav* 2005;84(1):53–63.

12. Pryor J, Patrick SW, Sundermann AC, Wu P, Hartmann KE. Pregnancy Intention and Maternal Alcohol Consumption. *Obstet Gynecol* 2017;129(4):727–733.

13. Anthony B et al. Alcohol-induced facial dysmorphology in C57BL/6 mouse models of fetal alcohol spectrum disorder. *Alcohol* 2010;44(7–8):659–671.

14. Boschen KE, Gong H, Murdaugh LB, Parnell SE. Knockdown of Mns1 Increases Susceptibility to Craniofacial Defects Following Gastrulation-Stage Alcohol Exposure in Mice. *Alcohol Clin Exp Res* 2018;42(11):2136–2143.

15. Kaminen-Ahola N et al. Maternal ethanol consumption alters the epigenotype and the phenotype of offspring in a mouse model.. *PLoS Genet* 2010;6(1):e1000811.

16. Klingenberg C et al. Prenatal Alcohol Exposure Alters the Patterns of Facial Asymmetry. *Alcohol* 2010;44(7–8):649–657.

17. Lipinski RJ et al. Ethanol-Induced Face-Brain Dysmorphology Patterns Are Correlative and Exposure-Stage Dependent. *PLOS ONE* 2012;7(8):e43067.

18. Whitten WK, Bronson FH, Greenstein JA. Estrus-inducing pheromone of male mice: transport by movement of air. *Science* 1968;161(3841):584–585.

19. Thomas KN et al. Paternal alcohol exposures program intergenerational hormetic effects on offspring fetoplacental growth [Internet]. *Frontiers in Cell and Developmental Biology* 2022;10.https://www.frontiersin.org/articles/10.3389/fcell.2022.930375. cited November 29, 2022

20. Chang RC et al. DNA methylation-independent growth restriction and altered developmental programming in a mouse model of preconception male alcohol exposure. *Epigenetics* 2017;12(10). doi:10.1080/15592294.2017.1363952

21. Chang RC, Wang H, Bedi Y, Golding MC. Preconception paternal alcohol exposure exerts sex-specific effects on offspring growth and long-term metabolic programming. *Epigenetics and Chromatin* 2019;12(1). doi:10.1186/s13072-019-0254-0

22. Thomas KN et al. Maternal background alters the penetrance of growth phenotypes and sex-specific placental adaptation of offspring sired by alcohol-exposed males. *FASEB J* 2021;35(12):e22035.

23. Spear LP, Heyser CJ. Is use of a cellulose-diluted diet a viable alternative to pair-feeding?. *Neurotoxicol Teratol* 1993;15(2):85–89.

24. Leeman RF et al. Ethanol Consumption: How Should We Measure It? Achieving Consilience between Human and Animal Phenotypes. *Addict Biol* 2010;15(2):109–124.

25. Thiele TE, Crabbe JC, Boehm SL. “Drinking in the Dark” (DID): a simple mouse model of binge-like alcohol intake.. *Curr Protoc Neurosci* 2014;68:9.49.1-12.

26. Naimi TS et al. Binge drinking among US adults.. *JAMA* 2003;289(1):70–75.

27. White AM, Kraus CL, Swartzwelder H. Many college freshmen drink at levels far beyond the binge threshold.. *Alcohol Clin Exp Res* 2006;30(6):1006–1010.

28. Truett GE et al. Preparation of PCR-quality mouse genomic DNA with hot sodium hydroxide and tris (HotSHOT). *Biotechniques* 2000;29(1):52, 54.

29. Carter RC et al. Fetal Alcohol Growth Restriction and Cognitive Impairment.. *Pediatrics* 2016;138(2). doi:10.1542/peds.2016-0775

30. Wozniak JR, Riley EP, Charness ME. Clinical presentation, diagnosis, and management of fetal alcohol spectrum disorder. *The Lancet Neurology* 2019;18(8):760–770.

31. Katsube M, Yamada S, Utsunomiya N, Morimoto N. Application of geometric morphometrics for facial congenital anomaly studies. *Congenit Anom (Kyoto)* 2022;62(3):88–95.

32. Zelditch ML, Swiderski DL, Sheets HD. *Geometric Morphometrics for Biologists: A Primer*. Academic Press; 2012:

33. Klingenberg CP. MorphoJ: an integrated software package for geometric morphometrics. *Mol Ecol Resour* 2011;11(2):353–357.

34. Attanasio C et al. Fine tuning of craniofacial morphology by distant-acting enhancers. *Science* 2013;342(6157):1241006.

35. Wiseman DN, Samra N, Román Lara MM, Penrice SC, Goddard AD. The Novel Application of Geometric Morphometrics with Principal Component Analysis to Existing G Protein-Coupled Receptor (GPCR) Structures. *Pharmaceuticals* 2021;14(10):953.

36. Geometric Morphometrics Tutorial | Sam Penrice [Internet]https://sampenrice.com/geometric-morphometrics-tutorial/. cited February 9, 2023

37. Roussos P, Mitsea A, Halazonetis D, Sifakakis I. Craniofacial shape in patients with beta thalassaemia: a geometric morphometric analysis. *Sci Rep* 2021;11(1):1686.

38. Rohlf FJ. tpsUtil. file utility program–Department of Ecology and Evolution, State University of New York at Stony Brook. *Search in* 2015;

39. Rohlf FJ. tpsDig, digitize landmarks and outlines, version 2.05. *Department of Ecology and Evolution, State University of New York at Stony Brook* 2005;

40. Perez KE, King-Heiden TC. Geometric Morphometrics as a Tool to Evaluate Teratogenic Effects in Zebrafish (Danio rerio) [Internet]. In: Félix L ed. *Teratogenicity Testing: Methods and Protocols*. New York, NY: Springer; 2018:373–391

41. Hammer O, Harper DAT, Ryan PD. PAST: Paleontological Statistics Software Package for Education and Data Analysis

42. Basnet BB et al. An anthropometric study to evaluate the correlation between the occlusal vertical dimension and length of the thumb. *Clin Cosmet Investig Dent* 2015;7:33–39.

43. Parnell SE et al. Maternal oral intake mouse model for fetal alcohol spectrum disorders: ocular defects as a measure of effect. *Alcohol Clin Exp Res* 2006;30(10):1791–1798.
